# Non-invasive Vagus Nerve Stimulation in Treatment of Disorders of Consciousness – Longitudinal Case Study

**DOI:** 10.3389/fnins.2022.834507

**Published:** 2022-05-06

**Authors:** Albertyna Osińska, Andrzej Rynkiewicz, Marek Binder, Tomasz Komendziński, Anna Borowicz, Antoni Leszczyński

**Affiliations:** ^1^Faculty of Psychology, University of Warsaw, Warsaw, Poland; ^2^Institute of Psychology, Jagiellonian University, Kraków, Poland; ^3^Department of Cognitive Science, Faculty of Humanities, Nicolaus Copernicus University in Toruń, Toruń, Poland

**Keywords:** vagus nerve stimulation, taVNS, disorders of consciousness, coma, unresponsive wakefulness syndrome (UWS), EEG, HRV

## Abstract

Neuromodulatory electroceuticals such as vagus nerve stimulation have been recently gaining traction as potential rehabilitation tools for disorders of consciousness (DoC). We present a longitudinal case study of non-invasive auricular vagus nerve stimulation (taVNS) in a patient diagnosed with chronic unresponsive wakefulness syndrome (previously known as vegetative state). Over a period of 6 months we applied taVNS daily and regularly evaluated the patient’s behavioral outcomes using Coma Recovery Scale – Revised. We also took electrophysiological measures: resting state electroencephalography (EEG), heart rate (HR) and heart rate variability (HRV). All these methods revealed signs of improvement in the patient’s condition. The total CRS-R scores fluctuated but rose from 4 and 6 at initial stages to the heights of 12 and 13 in the 3rd and 5th month, which would warrant a change in diagnosis to a Minimally Conscious State. Scores obtained in a 2 months follow-up period, though, suggest this may not have been a lasting improvement. Behavioral signs of recovery are triangulated by EEG frequency spectrum profiles with re-emergence of a second oscillatory peak in the alpha range, which has been shown to characterize aware people. However, sustained spontaneous theta oscillations did not predictably diminish, which most likely reflects structural brain damage. ECG measures revealed a steady decrease in pre-stimulation HR combined with an increase in HRV-HR. This suggests a gradual withdrawal of sympathetic and an increase in parasympathetic control of the heart, which the previous literature has also linked with DoC improvements. Together, this study suggests that taVNS stimulation holds promise as a DoC treatment.

## Introduction

### Promise of Transcutaneous Auricular Vagus Nerve Stimulation for Disorders of Consciousness

The prolonged disorders of consciousness (DoC) are one of the most severe outcomes of brain damage. They are also notoriously difficult to both diagnose and rehabilitate. For the latter, emerging techniques of brain stimulation have shown promise. With the view of prospective widespread use, of particular interest are especially the non-invasive methods which can be applied in hospital as well as home settings, e.g., transcranial direct current stimulation (tDCS) or low-intensity focused ultrasound pulse. Recently, encouraging results have come from stimulating the brain via the vagus nerve (see Briand et al., 2020 for an overview). While vagus nerve stimulation (VNS) is mostly known as an adjunct treatment for epilepsy (Ben-Menachem, 2002) and depression (Sackeim, 2001) as well as motor-control recovery after stroke (Dawson et al., 2016), studies are beginning to report improvements in patients with DoCs as well. Traditionally, this type of stimulation was done using a surgically implanted device [referred here as vagus nerve stimulation (VNS)]. However, comparable results can be achieved transcutaneously, simply by attaching an electrode to the left ear, where the current passes to the auricular branch of the vagus nerve [referred here as transcutaneous auricular vagus nerve stimulation (taVNS); Kraus et al., 2013; Capone et al., 2014]. The taVNS may theoretically be a safer option as it selectively stimulates the afferent vagus nerve fibers whereby minimizing potential cardiac risks of efferent fiber activation of the implanted VNS (Kreuzer et al., 2012; Chen et al., 2015; Hakon et al., 2020; Yap et al., 2020).

Vagus nerve (X cranial nerve) is the longest and most widespread of the cranial nerves (*vagus* in Latin means “wandering”). It is in fact a nerve pair (left and right), usually referred to in the singular. It runs from the brain through the face, thorax to the abdomen, where it innervates all the major internal organs. It contains parasympathetic fibers of a mixed type (80% afferent, relaying sensory information from the periphery to the brain; and 20% efferent, motor fibers, carrying impulses away from the central nervous system).

Auricular branch of the vagus nerve (Arnold’s nerve) is responsible for somatosensory innervation of the ear. Sensory fibers are located in the central part of the external ear, near the part of the auricle called Cymba Concha. Afferent neurons of the auricular vagus nerve reach the inferior vagal ganglion, and then stimulation is transmitted to the nucleus of the solitary tract in the brainstem. The solitary tract is the main receiver of stimulation from different branches of the vagus nerve and it has numerous output connections, e.g., with the locus coeruleus – this part of the reticular activating system is the main source of adrenergic incentive projections to cortical, subcortical and brainstem circuits. Locus coeruleus activation is probably responsible for many observed therapeutic effects of VNS and taVNS (Butt et al., 2020).

### Vagus Nerve Stimulation and Transcutaneous Auricular Vagus Nerve Stimulation in Disorders of Consciousness Treatment

To the best of our knowledge only seven studies so far have looked at vagus nerve stimulation in DoC patients, all reporting at least some encouraging findings. Two of them utilized the invasive, VNS technique, administered through a surgically implanted device, and five the non-invasive counterpart through stimulation of the auricular branch (taVNS), see Table 1 for details.

**TABLE 1:** A summary of VNS and tVNS studies on DoC patients.

	Participants and diagnoses	Time since injury	Stimulation site and device	Stimulation parameters	Stimulation protocol	Assessment	Outcomes
**VNS**							
Corazzol et al., 2017	1 UWS Age: 35 Etiology: Traumatic	15 years	Neck level, Cyberonics Inc.	0.25 mA/30 Hz/500 μs; gradually increased to 1.5 mA	30 s stimulation by 5 min rest Duration: 6 months	CRS-R, EEG, PET	CRS-R: 5 → 10 (but not sustained) EEG: ↑ rs theta band (4–7 Hz) power; sources in the DMN; global increase in mean wSMI over theta sources significantly correlated with CRS-R scores PET: ↑ occipito-parieto-frontal and basal ganglia regions ↑ thalamus
Xiang et al., 2020	10 MCS Age: 43.90 ± 15.79 Etiology: Traumatic: 4 Hemorrhage: 5 Ischemic-hypoxic: 1	7.16 ± 2.12 months Range: 5–11.5 months	G112, PINS Medical, Ltd.	0.1–0.3 mA/20–30 Hz/250 or 500 μs, gradually increased to 1.5 mA, then individual adjustments up to 3.5 mA	30 s stimulation by 5 min rest Duration: 6 months	CRS-R	1 month: no sig. total scores vs. baseline 3 months: sig. total scores vs. baseline (*p* = 0.0078) 3/10 – sig. improvement 2/10 – VNS-responders 5/10 – unresponsive to VNS Subscales: visual function more sensitive to VNS (*p* = 0.0156) 6 months: sig. total CRS-R scores vs. baseline (*p* = 0.0039) 1/10 –has emerged from minimally conscious state 2/10 – sig. improvement 3/10 – responsive 5/10 – unresponsive to VNS Subscales: sig visual vs. baseline (*p* = 0.0078) No diff. in CRS-R between 6- and 3-month follow-ups (*p* > 0.05)
**taVNS**							
Yu et al., 2017	1 UWS Age: 73 Anoxic: 1	50 days	Cymba concha (bilateral) Custom device	4–6 mA/20 Hz/<1,000 μs	Twice daily, for 30 min Duration: 4 weeks	CRS-R, fMRI	CRS-R baseline 6, at 4 weeks: 13, change UWS → MCS; new behaviors in motor and oromotor function ↑FC between posterior cingulate/precuneus and hypothalamus, thalamus, vmPFC, superior temporal gyrus ↓FC between posterior cingulate/precuneus and the cerebellum
Noé et al., 2020	6 UWS 8 MCS Age: 40.2 ± 16.1 Etiology: Traumatic: 7 Anoxic: 4 Hemorrhage: 3	12.1 ± 6.4 months	Left tragus Parasym ^®^ CE	1.5 mA/20 Hz/250 μs	30-min twice a day, 5 days a week Duration: 4 weeks + follow-up after 4 weeks	CRS-R	Responders (showing improvement in at least one item in CRS-R): 0/6 UWS 5/8 MCS – in all but one improvement occurred in the 4-week follow-up after taVNS Subscales: - Motor: 3/5 responders - Visual: 1/5 - >1 subscale: 1/5
Hakon et al., 2020	3 UWS 2 MCS Age: Median 46 Range: 21–80 Etiology: Traumatic: 5	Median: 41 days Range: 31–95 days	Cymba concha NEMOS ^®^	0.5 mA for the first 3 days, then 1 mA/25 Hz/250 μs	4 h daily, 30 s on/30 s off Duration: 8 weeks	CRS-R	3/5 patients showed improvement (>3 points); of these: UWS → EMCS (5→22) MCS → EMCS (10→23) UWS → MCS (3→6) Remaining patients: MCS → MCS (12 → 12) UWS → UWS (2 → 3)
Yu et al., 2021	7 UWS 3 MCS Age: 36 ± 15.3 Range: 19–73 Etiology: Hypoxic-ischemic Encephalopathy (HIE): 5 Traumatic: 2 Cerebral hemorrhage: 2 Brainstem hemorrhage: 1	78.5 ± 83.3 Range: 10–300	Cymba concha and cavity concha Custom device	4–6 mA/20 Hz/500 μs	30 min continuously, twice daily Duration: 4 weeks	CRS-R, GOS, fMRI	CRS-R: 5 responded to auditory stimuli (RtAS) 5 did not respond to auditory stimuli (nRtAS) GOS: Only RtAS got favorable prognoses after treatment (GOS > 2) fMRI RtAS: taVNS increased CBF of multiple brain regions nRtAS: taVNS increased CBF only in the left cerebellum

*CBF, cerebral blood flow; CRS-R, coma recovery scale-revisited; DMN, default mode network; EMCS, emergence of minimal conscious state; FC, functional connectivity; GOS, Glasgow Outcome Scale; MCS, minimal conscious state; UWS, unresponsive wakefulness syndrome, fMRI, functional magnetic resonance imaging; Hz, Hertz; mA, milliamperes; μs, microseconds; s, seconds; PET, positron emission tomography; taVNS, transcutaneous auricular vagus nerve stimulation (non-invasive, via an electrode attached to the ear); vmPFC, ventromedial prefrontal cortex; VNS, vagus nerve stimulation (via a surgically inserted implant); wSMI, weighted symbolic mutual information.*

Together these studies (presented in Table 1) suggest that although promising as a DoC treatment, taVNS may not hold equal promise for all. A study by Yu et al. (2021) presents an important step in determining the characteristics of responders. Out of 10 patients (seven UWS and three MCS) it turned out that only those who reacted to auditory stimuli during the CRS-R examination benefited from taVNS, as evidenced by behavioral and neuroimaging data.

The studies differ not only in patient populations and outcomes, but also devices used and electrode placement on the ear. Auricular branch of the vagus nerve (ABVN) has been shown to innervate the tragus and concha, in particular cymba concha (Peuker and Filler, 2002). Yet there is no consensus on which of these presents the optimal location for stimulation, e.g., Napadow et al. (2012) indicate the concha as the best site, while Kraus et al. (2013) suggest the anterior wall of the auditory canal. Different manufacturers have targeted different locations with their devices, i.e., NEMOS by tVNS Technologies stimulates the ABVN via the cymba concha, while Parasym by Parasym Health, via the tragus. taVNS stimulator can also be relatively easily assembled using transcutaneous electrical nerve stimulator (TENS) devices, commonly used for pain management, with suitable electrodes. Further research is needed to explore electrode placement and stimulation parameters to bring the greatest therapeutic effects to particular conditions.

In terms of outcome measures, previous studies have mostly focused on behavioral assessment, namely CRS-R evaluation. PET or fMRI have been rarely used, as their administration in vulnerable populations such as DoC patients requires highly specialist procedures (e.g., fMRI is carried out under sedation or anesthesia for which some patients may have contraindications). By contrast, EEG and ECG measures can also be taken at bedside and enable additional evaluation of the patient’s condition and treatment efficacy. Among the VNS studies in DoC so far only one measured EEG activity (Corazzol et al., 2017) and none of the taVNS interventions. Corazzol et al. (2017) revealed an increase in theta band (4–7 Hz) power, which previous work has indicated as reliably distinguishing MCS from UWS patients (Sitt et al., 2014). Spectra profiles of resting-state EEG have been correlated with DoC severity (Forgacs et al., 2017, 2020; see more in section “Discussion”), but this area has been unexplored yet among the studies of taVNS in DoC.

### Mechanism of Action

Because various etiologies can cause DoC, e.g., traumatic brain injury, anoxia, infection, and consciousness may dissociate from motor behavior, it is particularly challenging to pinpoint neural correlates of consciousness and model its recovery. Based on a review of interventional studies on pharmacological and electrical stimulation in patients with longstanding DoCs, as well as functional and structural neuroimaging literature, Schiff (2010) proposed a “Mesocircuit hypothesis” of consciousness recovery. According to the model, one of the key obstacles to functional recovery is widespread deafferentiation and disconnection of neurons located mainly in the central thalamus. As a result, the neuronal circuit involved in cortical activation is disturbed. What follows is that interventions involving stimulation of the thalamus should hold particular promise for DoC patients. In terms of the mechanism of taVNS in DoC recovery specifically, a dedicated “Vagal Cortical Pathway Model” has been proposed by Briand et al. (2020). This model describes both the direct and indirect pathways connecting the vagus nerve with, e.g., nuclei of the thalamus and proposes that taVNS stimulates the recovery through activation of the ascending reticular activating system (ARAS), the thalamus, the striatum and through re-establishment of the cortico-striatal-thalamic-cortical loop. It also proposes improvements in activity and connectivity within the default mode network (DMN) and the External Fronto-Parietal Network (ExN) as well as activation of the Salience Network (SN) (see Briand et al., 2020, for details). At a cognitive level of analysis, consciousness restoration through taVNS is consistent with an embodied, interoceptive model of the conscious self where the brain is assumed to constantly evaluate the signal from the physical body (Seth et al., 2012; Seth, 2013; Cleeremans et al., 2020; Paciorek and Skora, 2020; Yu et al., 2021). Stimulating the vagus nerve as the main pathway relaying visceral signals into the brain, therefore, offers a remarkable potential bottom-up approach for DoC therapy.

### Need for Multiple Sources of Evidence in Disorders of Consciousness Diagnosis

Finally, and perhaps fundamentally, consciousness itself remains notoriously hard to define and, consequently, diagnose. Wakefulness (arousal) and awareness have been assumed to be its two key components (Laureys, 2005; Briand et al., 2020). Wakefulness pertains to alertness or vigilance while awareness refers to the ability to interact with the environment or the self. Based on the degree of severity DoCs have been classified into: coma – absence of both wakefulness and awareness, UWS – intermittent periods of wakefulness with the absence of awareness of the environment or the self, MCS – variations in wakefulness and minimal, fluctuant but definite signs of awareness (as evidenced by, e.g., visual pursuit or command following). Currently the recommended diagnostic scale is the Coma Recovery Scale – Revised (Giacino et al., 2004), though a full diagnosis is not possible through bedside behavioral observation alone. That is because in DoCs behavior and neurophysiological evidence may dissociate. The best-known example is the study by Owen et al. (2006), in which functional magnetic resonance imaging (fMRI) showed that a patient diagnosed as UWS activated the predicted brain areas when asked to imagine playing tennis or moving around the home. Similarly, Bekinschtein et al. (2009) demonstrated electromyographic evidence of trace conditioning in patients diagnosed as UWS. Such learning involves a temporal gap between the conditioned and unconditioned stimuli and has been known to require explicit knowledge of the temporal contingency. Several independent ERP (event related potential) studies have shown semantic processing in UWS patients, including the presence of the N400 (Schoenle and Witzke, 2005), or reactions to patients’ own name, as evidenced by P300 (Perrin et al., 2006) or a stronger theta wave synchronization than other names (Fellinger et al., 2011). All these results therefore provide evidence for conscious processing not detected by behavioral assessment alone.

In light of this diagnostic challenge, to make as accurate assessment as possible, multiple sources of evidence are usually combined with behavioral evaluation, such as electroencephalography (EEG), functional magnetic resonance (fMRI) or positron emission tomography (PET). In addition, it is possible to use autonomic physiological indicators to assess the general homeostatic and psychophysical state of patients. Autonomic functions are usually regulated by the nuclei located in the brainstem and therefore can remain intact even when the brain is severely damaged. A fairly popular indicator is heart rate variability – HRV (Berntson et al., 1997). Based on the results of a number of studies carried out over the years, a neurovisceral integration model was proposed (Thayer et al., 2012), according to which the higher baseline level of HRV is the index of flexible control over behavior by cortical, subcortical and peripheral neural systems. DoC studies have shown higher levels of resting HRV in MCS than UWS patients, which means that the use of HRV indicators may increase the accuracy of the differentiation between UWS and MCS (Riganello et al., 2018) and possibly be used to detect awareness. The HRV has also been shown to be sensitive to DoC severity measured with Glasgow Coma Scale: HRV amplitude in both low (HRV-LF) and high (HRV-HF) frequency ranges was lower when GCS score was lower (Estévez-Báez et al., 2019).

To this body of literature we contribute here a longitudinal (6-month intervention with a 2-month follow-up), clinical case study of taVNS stimulation of a patient diagnosed with persistent UWS for 6 years following a traumatic brain injury. We combine multiple sources of evidence: behavioral (CRS-R) with neurophysiological analysis of the EEG signal and physiological processes evidenced by HRV indicators. We hypothesized that if transcutaneous auricular vagus nerve stimulation (taVNS) indeed affected the patient’s physical and psychological state, we would find improvement in behavioral responses on a Coma Recovery Scale – Revised (CRS-R), increase in resting HRV and in power of oscillations in the theta-alpha range (4–16 Hz) in resting state EEG.

## Materials and Methods

The research was carried out at the inpatient rehabilitation facility of the “Światło” (eng. “Light”) Foundation in Toruń, Poland, from June of 2019 to February of 2020. Ethical approval was obtained from the Ethical Committee at the Faculty of Psychology, University of Warsaw. Written informed consent to participate in this study and to publish the work was signed by the legal guardian of the patient.

### Patient

Our patient is a 28 years old woman who suffered traumatic brain injury 6 years prior to the study. She was diagnosed with persistent UWS and her CRS-R score prior to the study’s beginning was 4. Her condition had remained stable since the injury. She breathed independently via a tracheostomy tube and was fed through percutaneous endoscopic gastrostomy (PEG). Most of her time she spent in bed. Prior to participating in this study, the patient underwent a standard treatment for the patients with UWS/VS diagnosis, employed in the rehabilitation center. It included daily physical therapy, and 2–3 times a week multisensory stimulation, and speech therapy (training of orofacial reflexes). She was regularly consulted by the medical staff and received 24-h nursing support.

She was selected based on the following inclusion criteria: long time since brain injury (more than 2 years) to ensure any improvement was unlikely to be a result of spontaneous recovery, stable CRS-R results prior to the study, stable day/night rhythm, normal blood pressure level and ECG, lack of significant losses of cortical tissue, autonomic respiratory action without assistance, intact vagus nerve.

### Transcutaneous Auricular Vagus Nerve Stimulation

NEMOS ^®^ stimulator (tVNS Technologies, Erlangen, Germany) was used to administer taVNS. It comprises a small, portable stimulation unit with an intra-auricular epidermic electrode that is placed in the auricular tract with a contact point at the cymba concha. The settings were fixed at continuous 0.25-ms-duration monophasic square wave pulses at 25 Hz frequency, fixed 25 V voltage, 30 s on/30 s off. The current was changed systematically, starting at 0.2 mA and increasing the intensity by 0.1 mA every week up to 1.5 mA, following the protocol of a previous study (Corazzol et al., 2017). The stimulation was applied for 4 h a day, based on the device recommendations provided by the manufacturer for patients with epilepsy.

### Behavioral Measure

A Polish version of the CRS-R was used (Binder et al., 2017). CRS-R is currently the gold standard for DoC diagnosis and monitoring of recovery (Giacino et al., 2004). It consists of six subscales: auditory, visual, motor, verbal communication, and arousal. The maximum score is 23, less than 10 points usually corresponds to UWS. Low score is associated with basic reflexes, whereas high score indicates controlled, intentional behavior. Assessment was conducted weekly, right before the stimulation period, then for a 6-month daily stimulation period, as well as for 9 weeks after the stimulation was ended to assess and monitor any behavioral changes. It was conducted by one of two trained research students. Assessment sessions were recorded and discussed to assure the measurement reliability.

### Electrophysiological Measure

#### Electroencephalography Measurement

The EEG resting-state recording was performed using 64-electrode Active Two (BioSemi, Amsterdam, Netherlands), with a 10–20 system headcap. Electrooculography (EOG) signal was acquired using four electrodes located above and below the right eye and in the external canthi of both eyes. Two reference electrodes were attached on the left and the right mastoid and recorded in parallel. Two electrodes specific to Active Two system, namely “CMS” (common mode sense) and “DRL” (driven right leg), were placed between “POz” and “PO3” and “POz” and “PO4,” respectively. Recording duration was 10 min, eye-opening was maintained during recording. EEG signal was sampled at 1,024 Hz. The recording took place in the isolated room, with the patient sitting in bed in a reclining position, or sitting on the wheelchair in a comfortable position.

The EEG was performed six times with the mean interval of 49 days (range 36–61 days). The first measurement preceded the start of the taVNS stimulation by 1 month, the next four were performed during stimulation and the last one 56 days after taVNS stimulation protocol was completed. The relevant chronological information is presented in Table 2.

**TABLE 2:** Chronological information about subsequent EEG measurements.

Measurement ID	Relative day
EEG 1	10
EEG 2	66
EEG 3	95
EEG 4	131
EEG 5	192
EEG 6	248

*The days are calculated relative to the day when the stimulation protocol was started.*

#### Electroencephalography Analysis

The off-line preprocessing of EEG data was performed with Brain Vision Analyzer 2.0 software (Brain Products, Gilching, Germany). Noisy channels were interpolated with a spline method, data was filtered using high-pass and low-pass filters at 1–50 Hz range (IIR Filter, Zero phase shift Butterworth filter; order 8), and downsampled to 256 Hz. Raw data inspection excluded massive artifacts before the segmentation, whereas blink and eye movement artifacts were corrected using Independent Component Analysis (ICA). Data was further re-referenced to the common average. Next, signal from each channel was segmented into non-overlapping 2 s intervals, with the noisy segments excluded from the analysis using semi-automatic mode with the following criteria: amplitude limits −150 μV to 150 μV; 150 μV maximum allowed difference in intervals over 200 ms; maximal voltage step of 75 μV/ms. Fast Fourier Transform (FFT, 10% Hanning window, 0.5 Hz resolution) was calculated on the remaining segments and averaged, each channel separately. Then, the single centro-parietal region channel was constructed by averaging the signal from the electrodes “Cz,” “CPz,” “Pz,” “CP3,” “CP1,” “P3,” “P1,” “CP2,” “CP4,” “P2,” and “P4.” This region was used to calculate the frequency of the highest peak in the EEG spectrum. We decided to exclude other channels due to the frequent occurrence of artifacts of various origins precluding reliable analysis of EEG signal.

The averaged EEG spectra were parameterized using FOOOF algorithm (version 0.1.3). The fooof_mat wrapper (version 0.1.1, Donoghue et al., 2020) was used to analyze data within the MATLAB environment (MathWorks Inc., version 2019b). The FOOOF algorithm transforms the EEG signal spectrum into a set of parameters consisting of exponential two-parameter aperiodic components as well as a series of Gaussian peaks described by parameters referring to their center frequency, amplitude and bandwidth. The goodness of fit metrics are represented by *R*^2^ of the model fit and an error estimate. For the current analyses the default settings for the algorithm were as follows: peak width limits = 2–6, maximal number of peaks = 4, minimum peak height = 0.111, peak threshold = 2 and fixed aperiodic mode. Power spectra were parameterized across frequency range 2–45 Hz.

Based on FOOOF algorithm results, for each EEG measurement in the averaged signal from the centro-parietal region we obtained a Primary Peak Frequency (MaxPeak1) and Secondary Peak Frequency (MaxPeak2) parameters, which were the center frequencies of the peaks with the highest and second highest amplitude found within the search range 3–14 Hz (extending across theta and alpha EEG frequency bands).

#### ECG Measurement

For ECG data acquisition we used BITalino portable toolkit (Batista et al., 2019) and the OpenSignal (r)evolution software. We have used a specific arrangement of electrodes, which in bedside conditions provides comfort and does not require removing the upper part of clothing, and at the same time provides a clear shape of the QRS complex in the recorded ECG. Two active electrodes were attached on the sides of the chest and an inactive electrode was attached at the lower part of the sternum. Self-adhesive Ag/AgCl electrodes and standard ECG jelly were used. Raw ECG signal was transmitted to a portable computer via bluetooth and was visible online in the OpenSignal window. This hardware configuration ensured an appropriate safety standard for measurements as well as an ongoing control of the signal quality.

#### ECG Analysis

ECG was analyzed in two 5-min epochs – before the start (pre-stimulation) and after the stop (post-stimulation) of a taVNS session. The raw electrocardiogram for each epoch was visually inspected and corrected if necessary. Manual signal correction consisted only in shifting the boundaries of the measuring epoch so that it included the smallest number of artifacts and disturbances. Nevertheless, the correctional shift never exceeded 1 min.

Despite all efforts, we found some epochs with extremely disturbed signal and eliminated them from analysis. This is why the data sometimes contains recordings not from both, but only from the initial or only from the final epoch.

Epochs were cropped and corrected in the MATLAB environment. The same application was used to identify R-waves using a method based on the analysis of the shape of the first derivative of the ECG signal. Then inter-beat intervals (IBI) were calculated. For further analysis we used Kubios HRV (Tarvainen et al., 2014) with a built-in artifact correction algorithm (medium threshold) to reduce the impact of missed and extra beats on HRV. The IBI series was also detrended. As an index of HRV we used the FFT-based power spectrum. For our purpose only the high frequency range of the HRV (HRV-HF) was usable, so we analyzed the range from 0.15 to 0.4 Hz.

### Procedure

The research was carried out at the inpatient rehabilitation facility of the “Światło” (eng. “Light”) Foundation in the period from June of 2019 to February of 2020. We assumed that the tests would take place in the following cycle: (a) initial CRS-R measurement before taVNS started, (b) initial EEG measurement before taVNS started, (c) taVNS every day, (d) ECG measurement every other day before and after taVNS session, (e) CRS-R measurement once a week, (f) EEG measurement once a month. The entire research program took about 7 months including 6 months of taVNS. Due to unpredictable fluctuations in the health condition of the patient as well as equipment failures and measurement artifacts, the frequency of interventions and measurements sometimes fell below the assumed level. In total, we managed to complete over 100 taVNS sessions, 6 EEG measurements, 37 sets of ECG recordings (before and after stimulation) and 30 CRS-R measurements.

A single taVNS session lasted about 4 h and was always performed continuously at the same time of the day. If there was an ECG measurement on a given day, a trained student researcher connected the ECG electrodes to the patient’s chest and checked the correctness of the recorded signal on the computer monitor. The ECG signal was recorded for 11–12 min – the final 5-min period was used as a pre-stimulation level. Then the taVNS electrode was applied to the patient’s left ear and the stimulator was turned on. At the end of the 4-h session the researcher turned the stimulator off and started ECG recording. The ECG signal was measured for 11–12 min – the initial 5-min period immediately after taVNS was used as a post-stimulation level.

For safety precautions of the project, every day, when taVNS stimulation was switched on the patient was constantly accompanied by a trained student researcher who watched for any signs of patient discomfort and monitored stimulation stability. Stimulation was paused when the medical staff performed routine daily care activities.

## Results

### Behavioral Data

**FIGURE 1:**
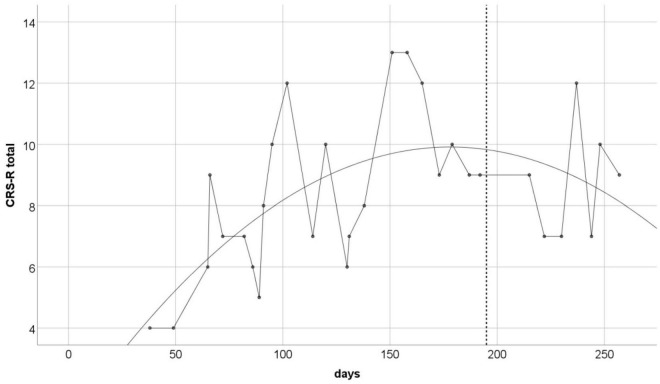
The CRS-R total scores over the study duration. Solid line is the best fitted non-linear regression curve (*r*^2^ = 0.36, *p* < 0.01). Vertical dashed line indicates the day of the last taVNS session.

The CRS-R measurement was performed approximately every 7 days throughout the 220-day study period. Obtained data creates a time series with irregular distances between measurements (Figure 1). Total scores ranged from 4 to 13 points (*M* = 8.4, SD = 2.46). To illustrate the trend of changes in the measurements over time, we used the regression model. As the non-linear trend of changes is also possible in our conditions, we decided to test linear as well as quadratic models of regression. Both were statistically significant, but the quadratic equation provided a better fit (*r*^2^ = 0.36; *p* < 0.01), than the linear one (*r*^2^ = 0.17; *p* < 0.01). The best fitted regression model is shown on Figure 1.

The total CRS-R score is the sum of scores for six subscales. Standard deviation in each of them shows that the Auditory Functions Scale (SD = 0.97) and Visual Functions Scale (SD = 1.14) had the greatest impact on the observed fluctuations in the overall results. In other subscales standard deviations did not exceed 0.6.

### Electrophysiological Data

**FIGURE 2:**
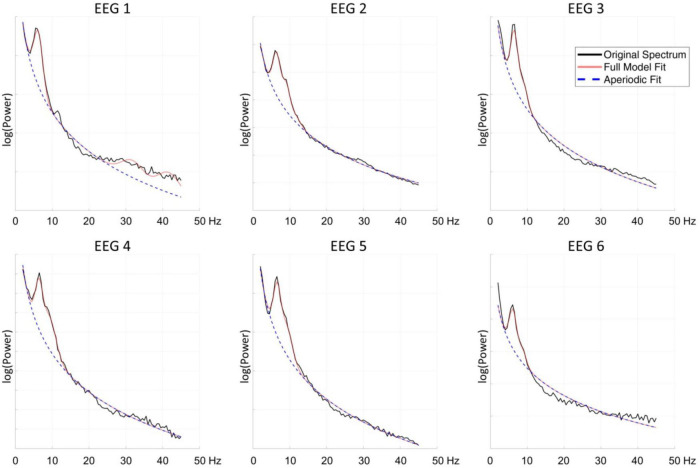
The spectral profiles of all six measurements from the centro-parietal channel. Black line indicates the original spectrum, the red line the fitted function obtained with FOOOF algorithm, and the blue dashed line the fitted aperiodic component.

**FIGURE 3:**
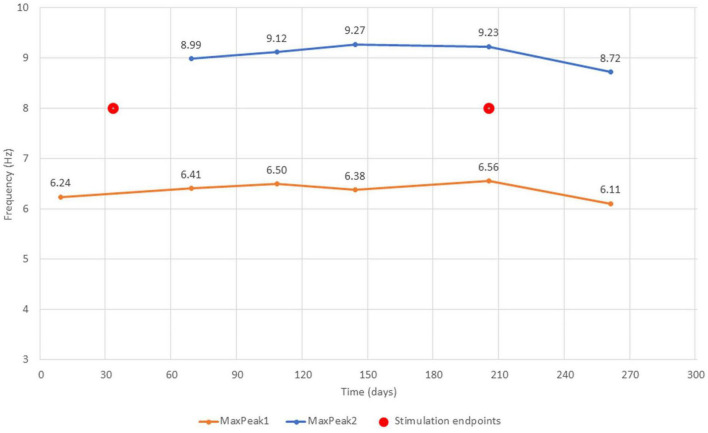
The changes of MaxPeak1 and MaxPeak2 center frequencies across all six measurements. Captions accompanying the score points indicate the center frequency score. Stimulation endpoints (red circles) indicate the first and the last day of stimulation protocol.

The FOOOF algorithm decomposition of EEG signal from the centro-parietal channel in all six measurements identified peaks within 3–14 Hz search range indicating the presence of spontaneous oscillations within the resting-state EEG signal. The analysis revealed spectral profiles that underwent both qualitative as well quantitative changes over the course of six measurements. In all measurements we observed theta oscillations (MaxPeak1) that were centered around 6.37 Hz (range: −0.26/+0.19 Hz, SD = 0.17). The estimated amplitude of the MaxPeak1 was relatively stable, averaging at 0.68 μV^2^ (range: −0.15/+0.12, SD = 0.09). An important qualitative change was the occurrence of the second, smaller peak within the alpha band range. The mean frequency of this second oscillatory peak (MaxPeak2) was 9.07 Hz (range −0.35/+0.20 Hz, SD = 0.22). The MaxPeak2 was not detected by FOOOF algorithm in measurement administered before the taVNS program started. It appeared in the second measurement and its presence was maintained through the remainder of the measurements. The amplitude of the second peak was smaller and more variable, the average value was 0.36 μV^2^ (range: −0.13/+0.17, SD = 0.12). The full model goodness-of-fit was high in all measurements, except the last one in which it decreased due to the presence of unmodeled high frequency noise in the signal. The detailed information about the oscillatory peaks is presented in Table 3. Figure 2 shows the spectral profiles from all measurements, and Figure 3 the changes of the center frequencies of MaxPeak1 and MaxPeak2.

**TABLE 3:** The detailed information about the MaxPeak1 and MaxPeak2.

Meas. ID	MaxPeak1 center frequency (Hz)	MaxPeak1 amplitude (μV^2^)	MaxPeak2 center frequency (Hz)	MaxPeak2 amplitude (μV^2^)	*R* ^2^	Error estim.
EEG 1	6.24	0.70	Not found	Not found	0.9923	0.0411
EEG 2	6.41	0.79	8.99	0.53	0.9985	0.0222
EEG 3	6.50	0.73	9.12	0.28	0.9903	0.0498
EEG 4	6.38	0.53	9.27	0.34	0.9959	0.0267
EEG 5	6.56	0.69	9.23	0.40	0.9969	0.0286
EEG 6	6.11	0.61	8.72	0.23	0.9758	0.0640

### ECG

The ECG was recorded twice – before and after a taVNS session. Each segment lasted 5 min and was used to calculate mean HR and HRV-HF levels. Over the entire measurement period of 167 days, we collected 37 series covering the pre-stimulation and the post-stimulation segments. Our intention was to take measurements every 2 days. Nevertheless, the measuring equipment we used had two major disadvantages. The first was battery power. In the absence of a battery charge indicator, it was easy to overlook that it was only slightly charged. The second disadvantage is data transmission via Bluetooth, which was sometimes interrupted for unknown technical reasons. Of course, both of the above solutions also have a major advantage – ensuring patient safety. Thus, due to these technical problems and temporary health problems (e.g., viral infections) or physiological state (e.g., blood pressure and temperature changes during menstruation), the frequency of measurements was variable and therefore their time points are not distributed evenly.

**FIGURE 4:**
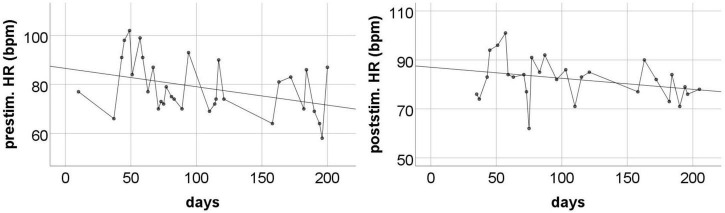
The pre-stimulation **(left panel)** and post-stimulation **(right panel)** HR levels from all measurements during the entire program period. Solid line is the best fitted regression line (mathematical description in Table 4).

The changes in HR during the entire measurement period showed quite a large variance – the HR level was moderately low on some days, and accelerated considerably on others, reaching sometimes over 100 beats per minute (Figure 4). The post-stimulation HR level was almost always higher than the pre-stimulation level – the average difference was 5.17 bpm (SD = 7.87). Nevertheless, a systematic decrease of HR was observed throughout the measurement period. Regression analysis showed that only pre-stimulation time series can be approximated by a line with a statistically significant negative coefficient (Table 4).

**TABLE 4:** Regression coefficients for HR levels (linear regression model was used).

	Reg. coeff.	*R* ^2^	*p*
Pre-stimulation level	−0.08	0.14	0.04
Post-stimulation level	−0.05	0.09	0.12

**FIGURE 5:**
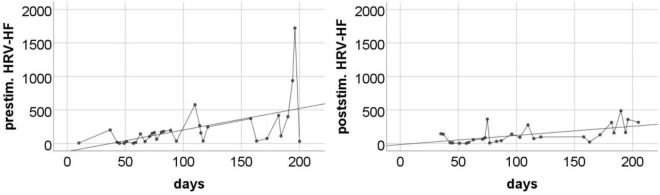
The pre-stimulation **(left panel)** and post-stimulation **(right panel)** HRV-HF power levels from all measurements during the entire program period. Solid line is the best fitted regression line (mathematical description in Table 5).

Changes in HRV-HF level also showed limited stability over the course of subsequent measurements. However, similarly to HR, these changes showed a systematic trend throughout the entire measurement period (Figure 5). The analysis showed that both pre-stimulus and post-stimulus changes can be approximated with regression lines with statistically significant positive coefficients (Table 5).

**TABLE 5:** Regression coefficients for HRV-HF levels (linear regression model was used).

	Reg. coeff.	*R* ^2^	*p*
Pre-stimulation level	3.22	0.28	0.002
Post-stimulation level	1.36	0.35	0.001

### Additional Observation

After less than 3 months of daily taVNS our patient began menstruating, which had not happened since the brain injury. This is likely to be the result of the intervention since the gonads are innervated by the vagus nerve. The fact that it appeared during the stimulation period is perhaps less crucial than the fact that it remained after the intervention finished, even if only in an on-again, off-again fashion. This implies that the intervention brought a durable change to our patient’s physiology.

## Discussion

In our study, we applied taVNS daily for 6 months to a patient diagnosed with UWS. Despite the fact that the measurement results were characterized by high instability, we observed a systematic increase of the total CRS-R score, which summarizes the behavioral indicators of consciousness. From the initial score of 4–6 points, the patient progressed to 8–10 points after about 100 days of stimulation, and occasionally the total CRS-R score increased even to 13. This means that the initial UWS diagnosis could be changed to MCS or even MCS+. Behavioral changes that were responsible for this effect were concentrated mainly in visual and auditory functions – the highest variance in results was observed in these two scales of the CRS-R. In the remaining scales, i.e., in motor and oromotor/verbal activity, communication and arousal, the observed changes over time had a much smaller range, so the results were more stable. This is in line with previous reports showing that first signs of spontaneous progression from UWS to MCS usually appear in visual and sometimes also in auditory functions (Bagnato et al., 2017; Bareham et al., 2019).

The apparent improvement in our patient’s functioning, however, did not seem to last. As the CRS-R evaluations continued to be performed for over 50 days after the end of the taVNS intervention, we noticed that the total score, as well as the regression trend, stopped rising. Still, a definite conclusion that the patient’s improvement was only temporary would be premature, as we could only perform these post-intervention evaluations for a limited, quite short, period of time (due to the breakout of the COVID-19 pandemic). CRS-R performed on our patient 1.5 years after the end of our study also revealed significant fluctuations in the total score ranging from 5 (UWS) to even 13 (MCS+). Observations made during testing showed that the patient obtained better results when the test was performed in a sitting position than when lying down in bed, which may be associated with changes in arousal level (Bareham et al., 2019). However, such changes were not demonstrated on the CRS-R arousal scale.

Multiple EEG registration also allowed us to observe interesting effects. We found prominent oscillations in the theta band (average frequency 6.73 Hz) in all six EEG measurements. Interestingly, on the second measurement, 36 days after the beginning of the taVNS stimulation, the spectral profile of the EEG signal changed and FOOOF decomposition suggested that another 9 Hz oscillation in the alpha band was present in the EEG signal. That second oscillatory component was visible until the last measurement. Moreover, between the first and the second measurement we also observed a notable change in the total CRS-R score which increased from 6 to 9 points and during the next four measurements, the total score sometimes even reached the level of 12–13.

On the basis of studies with post-anoxic patients (Forgacs et al., 2017, 2020) have proposed a classification of spectral profiles of resting-state EEG signal associated with various types of thalamocortical dysfunction. They have distinguished four main types of spectral profiles. The first, A-type is dominated by aperiodic activity with the highest power contained within the delta range and apparent 1/f pattern of the signal variability. A-type indicates complete loss of neural networks integrity, without any significant contribution of thalamic nor cortical function. Over the course of recovery another spectral profile may become visible – with significant presence of oscillations within the theta range, indicating only spontaneous cortical activity without thalamic output. In the third division – C-type –spontaneous theta oscillations are accompanied by beta oscillations in the frontal regions. That pattern, according to Forgacs et al. (2017) represents low thalamic activity appearing in bursting mode, and local cortical disinhibition. Finally, the D-type of spectral profile with prominent alpha and beta activity is an indicator of a normal cortical function, and a high, tonic level of thalamic activity. In our study we have observed an apparent transition from the B-type to the D-type of spectral activity. We have not observed the intermediate C-type, probably due to the limitations of our EEG analysis methods. The beta oscillations characteristic of the C-type activity are observed in the frontal regions, and we were not able to obtain artifact-free EEG signal from those locations, thus we are not in position to ascertain the presence of that spectral profile in our subject. However, contrary to Forgacs et al. observations, we have not observed diminishing of spontaneous theta activity in our subject, and these oscillations were present during the whole experiment duration. Thus the spectral pattern observed in the second and later measurements can be viewed as a mixture of a B-type and D-type of resting-state activity. However, we interpret the prolonged presence of theta oscillations as an indication of possible sustained structural damage to the thalamo-cortical system visible in the signal from the centro-parietal region. One may not exclude the hypothesis that the uncertain and variable diagnosis of the patient, as assessed by the repeated CRS-R administration, might have been caused by the coexistence of these two types of prominent neuronal activity. While the presence of local alpha-range oscillations within the centro-parietal region might demonstrate at least partial recovery of normal cortical function, the second theta-range oscillations might be related to the EEG slowing caused by structural brain damage (Synek, 1988). The available three head CT scans acquired within 1 month since the traumatic incident suggested relatively minor lesions of the hemispheres, the first, of hemorrhagic origin, spanning the left lentiform nucleus, as well as lesions of posterior forceps and posterior part of the corpus callosum. The brain stem lesions were also detected, yet they were difficult to the detailed assessment due to the limited CT resolution. We surmise that this relative sparing of cerebral hemispheres and thalamus might have been a factor facilitating the positive response to tVNS stimulation. However, this conclusion is limited by the fact that the CT scans have been acquired more than 4 years before the tVNS treatment, that later neuronal degeneration might have enlarged the territory of the telencephalic damage.

The similar relation between spectral oscillatory dynamics and condition of the patient was observed in other studies, yet only Forgacs et al. made an attempt to systematize the observed spectral profiles. For example (Lechinger et al., 2013) have observed a strong correlation between dominant posterior oscillatory activity within delta-theta-range and total CRS-R score. Relative increase in delta power, as well as decrease in alpha frequency was more often observed in patients with UWS diagnosis than with MCS in a study by Lehembre et al. (2012). Similar connections between behavioral and spectral measures in the delta-theta-alpha range were observed in a large study of Sitt et al. (2014), and in our study by Drążyk et al. (2022) involving 53 DoC patients. All these studies point at the presence of higher oscillations in alpha range as signs of recovery of consciousness, and at the same time the observed shift of the dominant frequency toward lower frequencies in the theta range was evidenced as a possible marker of disorganized brain dynamics due to sustained functional and/or structural damage.

The systematic ECG measurements showed that almost always post-stimulation HR level was higher than pre-stimulation level. Thus, it seems that the stimulation itself as well as the accompanying treatments could evoke some kind of sympathetically driven physiological arousal. Most likely, this is not a direct result of vagal nerve stimulation, because taVNS either does not cause changes in autonomic arousal (Couck et al., 2017) or causes a decrease in sympathetic arousal and a slower HR (Clancy et al., 2014). In our opinion, the acceleration of post-stimulation HR was rather the effect of mild discomfort associated with electrostimulation or possibly social stimulation related to the presence and activities performed by the student researcher. For this reason, the electrophysiological indicators recorded during the pre-stimulation sessions were more useful for us because they were devoid of situational artifacts.

The pre-stimulation level of HR showed large fluctuations over time, but a statistically significant downward linear trend was visible throughout the entire measurement period. This means that over the course of the study, we observed a systematic decrease in the tonic level of sympathetic arousal. This effect is consistent with the observations made by Clancy et al. (2014). It should be emphasized that the pre-stimulation level of HR was often high, reaching even 90–100 beats per minute in the initial months. This indicates the dominance of the sympathetic system in the regulation of the heart. Thus, the observed long-term decrease in HR should be understood as a gradual withdrawal of sympathetic stimulation and an increase in parasympathetic control. This interpretation is in line with our HRV-HF observations. Resting HRV-HF remained fairly low for the initial few weeks after starting the taVNS program. Later, on some days, this level increased significantly, and in the last period of stimulation, it sometimes reached very high. Regression analysis revealed a clear increasing trend. This is not a short-term reactive effect, as the trend was revealed in the resting data collected each day before the stimulation was started. Thus, we observed a long-term effect, which is most likely a consequence of relatively constant and stabilized regulatory processes.

Increase in resting HRV-HF levels is often associated with neurovisceral integration (Thayer et al., 2012), which in healthy people is an indicator of flexible control over behavior. According to the authors of this model, a high level of parasympathetic heart control means a state in which visceral systems are an important source of information about the body’s response to specific environmental influences. So it is an element of awareness of the environment. Some studies directly show that a higher level of HRV-HF in people with DoC often corresponds to a higher level of consciousness on the CRS-R (Riganello et al., 2018). Thus, an increase in the level of HRV-HF may be a signal of cognitive improvement, which cannot be overestimated in DoC patients. The research revealed that the level of resting HRV-HF shows a positive correlation with the capability of brain-computer interface usage (Kaufmann et al., 2012). Such effects constitute a potential direction for future research. If it could be confirmed that tVNS is able to raise the level of HRV-HF, the question arises whether this stimulation can increase the cognitive abilities of DoC patients large enough to make even minimal contact with them.

## Conclusion

We observed three positive phenomena accompanying our interventions. First, the behavioral indices of consciousness gradually increased throughout tVNS use. These indices stopped rising after stimulation was interrupted. Secondly, over the course of several months of stimulation, the EEG power in the range of the alpha wave gradually increased, which may be an indicator of a slight neural networks reintegration and strengthening of cortical activity. And thirdly, the systematic intervention was also accompanied by psychophysiological changes, i.e., the resting HR level gradually decreased and the HRV-HF level increased. The changes were not large, but visible in the form of long-term trends. They may be a sign of the increasing influence of the parasympathetic system in autonomic space, which usually helps to improve environmental consciousness.

However, it is clear that the case study does not provide conclusive evidence that the observed electrophysiological and behavioral effects are a consequence of tVNS. It is possible that the actual effect was due to social and physical stimulation during daily interventions, e.g., touch, sounds, changes in light intensity, etc. Thus, the phenomena described above require confirmation in studies with other patients (between-subject comparisons) or with the phase during which tVNS is sham (within-subject comparisons). In this case, the procedure will not be technically simple, because we observed changes in indicators after long-term exposure (at least several weeks of systematic interventions for 4 h a day). This period would extend even more after including the sham phase and some DoC patients may drop out of the program due to changes in physiological parameters (e.g., blood pressure spikes due to tVNS). Nevertheless, such trials could provide a clearer answer to the question of the benefits of using tVNS in the treatment of DoC patients.

## Data Availability Statement

The datasets presented in this article are not readily available because the datasets cannot be anonymized. Requests to access the datasets should be directed to the corresponding author.

## Ethics Statement

The study was reviewed and approved by the Research Ethics Committee at the Faculty of Psychology, University of Warsaw. The written informed consent to participate in this study as well as consent for the publication of any potentially identifiable images or data included in this article was obtained from the legal guardian of the patient.

## Author Contributions

AO: overall concept of the study, project coordination, ethics application, and article write-up (Introduction). AR: design of the ECG part of the project, ECG data analysis, and article write-up (Introduction, Results, and Discussion). MB: design of the EEG part of the project, EEG data collection and analysis, and article write-up (Results and Discussion). AB and AL: student researchers responsible for taVNS application, CRS-R, ECG, and EEG data collection. TK: project supervision, concept consultation, and data collection coordination. All authors contributed to the article and approved the submitted version.

## Conflict of Interest

The authors declare that the research was conducted in the absence of any commercial or financial relationships that could be construed as a potential conflict of interest.

## Publisher’s Note

All claims expressed in this article are solely those of the authors and do not necessarily represent those of their affiliated organizations, or those of the publisher, the editors and the reviewers. Any product that may be evaluated in this article, or claim that may be made by its manufacturer, is not guaranteed or endorsed by the publisher.
